# Delivering Guideline-Concordant Care for Patients With High-Risk HPV and Normal Cytologic Findings

**DOI:** 10.1001/jamanetworkopen.2024.54969

**Published:** 2025-01-17

**Authors:** Jasmin A. Tiro, Jacquelyn M. Lykken, Patricia M. Chen, Cheryl R. Clark, Sarah Kobrin, Jessica Chubak, Sarah Feldman, Claudia Werner, Steven J. Atlas, Michelle I. Silver, Jennifer S. Haas

**Affiliations:** 1 Department of Public Health Sciences, Division of the Biological Sciences, The University of Chicago, Chicago, Illinois; 2 Peter O’Donnell School of Public Health, University of Texas Southwestern Medical Center, Dallas; 3 Division of General Internal Medicine, Massachusetts General Hospital, Harvard Medical School, Boston; 4 Division of General Internal Medicine and Primary Care, Brigham and Women’s Hospital, Boston, Massachusetts; 5 Healthcare Delivery Research Program, Division of Cancer Control and Population Sciences, National Cancer Institute, Rockville, Maryland; 6 Kaiser Permanente Washington Health Research Institute, Seattle; 7 Division of Obstetrics Gynecology and Reproductive Biology, Brigham and Women’s Hospital, Harvard Medical School, Boston, Massachusetts; 8 Department of Obstetrics and Gynecology, University of Texas Southwestern Medical Center, Dallas; 9 Parkland Health, Dallas, Texas; 10 Department of Surgery, Washington University School of Medicine, St Louis, Missouri

## Abstract

**Importance:**

As US health care systems shift to human papillomavirus (HPV)–based cervical cancer screening, more patients are receiving positive high-risk non–16/18 genotype HPV results and negative for intraepithelial lesion or malignancy (NILM) cytological findings. Risk-based management guidelines recommend 2 consecutive negative annual results to return to routine screening.

**Objective:**

To quantify patterns of surveillance testing and associated outcomes for patients after an HPV-positive results and NILM cytologic findings.

**Design, Setting, and Participants:**

This cohort study analyzed patients in the METRICS (Multi-level Optimization of the Cervical Cancer Screening Process in Diverse Settings and Populations) cohort of the PROSPR II (Population-Based Research to Optimize the Screening Process) Cervical Consortium. Population-based data were obtained from 3 diverse health care systems (Mass General Brigham [MGB] in Massachusetts, Kaiser Permanente Washington [KPWA] in Washington, and Parkland Health [PH] in Texas) in the METRICS cohort. Participants were patients aged 21 to 65 years who received an HPV-positive (non-16/18 or pooled genotypes) result and NILM cytologic finding from January 2010 to August 2018 and were followed up through December 2019. Data analyses were performed between April 2021 and November 2024.

**Main Outcomes and Measures:**

Test receipt and outcomes delivered within 16 months after the index result (round 1 surveillance).

**Results:**

The final sample across the 3 health care systems comprised 13 158 female patients (3228 Hispanic or Latine [24.5%], 1990 non-Hispanic African American or Black [15.1%], 749 non-Hispanic Asian [5.7%], and 6559 non-Hispanic White [49.8%] individuals). Sociodemographic characteristics varied by site, with more non-Hispanic White (2277 [63.7%] and 4061 [61.2%]) and commercially insured patients (3137 [87.8%] and 4365 [65.7%]) at KPWA and MGB, and more Hispanic or Latine (1664 [56.5%]) and uninsured patients (2352 [79.9%]) at PH. During round 1 surveillance, 43.7% of patients were tested, of whom 18.2% (2394) had HPV-negative results and NILM cytologic findings and 25.5% (3351) had abnormal results. Many patients remained in the cohort and were untested through round 1 surveillance (overall: 49.4% [6505]; across sites: 39.0% [1395] to 69.4% [2043]), while fewer exited the cohort (overall: 6.9% [908]; across sites: 0.2% [12] to 24.6% [879]). Groups with lower odds of timely testing were younger adults (aged 25-29 vs 30-39 years: adjusted odds ratio [AOR], 0.65; 95% CL, 0.53-0.81), non-Hispanic African American or Black compared with non-Hispanic White patients (AOR, 0.78; 95% CL, 0.68-0.89), and those with Medicaid compared with commercial insurance (AOR, 0.81; 95% CL, 0.72-0.91), while those with a primary care clinician were more likely to have timely testing (AOR, 1.44; 95% CL, 1.21-1.70). Cancer was diagnosed in 10 patients (0.2%) untested in round 1 surveillance compared with 0 cancers in those with an HPV-negative results and NILM cytologic findings.

**Conclusions and Relevance:**

This cohort study found that among patients with HPV-positive results and NILM cytologic findings, less than half received a surveillance cotest during the guideline-recommended time frame. Health care systems should monitor annual surveillance and gather evidence on interventions to optimize the delivery of surveillance testing.

## Key Points

**Question:**

Among patients with positive high-risk non–16/18 genotype human papillomavirus (HPV) results and negative for intraepithelial lesion or malignancy (NILM) cytologic findings, how many received care in accordance with risk-based cervical cancer management guidelines?

**Findings:**

In this cohort study of 3 health care systems with 13 158 patients with an HPV-positive results and NILM cytologic findings, only 43.7% of patients retested within 16 months of receiving the result. There was wide variation in timely surveillance testing across cohort sites, with 10 invasive cervical cancers and 54 in situ cancers subsequently detected among those who were untested.

**Meaning:**

The findings suggest that given the suboptimal surveillance for patients with HPV-positive results and NILM cytologic findings, better monitoring of annual surveillance delivery is needed.

## Teaser

This cohort study examines adherence to cervical cancer testing for patients with non–16/18 genotype human papillomavirus (HPV) after receipt of initial abnormal cervical cancer result.

## Introduction

Guidelines are intended to increase delivery of the best care to the most people.^1,2^ Achieving this goal requires both well-founded guidelines and feasible implementation. The 2019 American Society for Colposcopy and Cervical Pathology (ASCCP) guidelines for managing abnormal cervical cancer screening results are designed to “ensure equal management for equal risks” and balance screening benefits with the harms of overtesting and treatment.^3^^(p103)^ A diverse set of stakeholders, including clinical specialty organizations and patient advocacy groups, was involved in the consensus process during guideline development.^4^ While these guidelines were based on the best available evidence and have an admirable goal, the feasibility of implementation in routine care is unclear. Uneven implementation could have adverse outcomes and perpetuate cervical cancer disparities.

To apply risk-based guidelines correctly, 2 elements are essential: (1) data on screening history and current risk factors, and (2) continued engagement with health care systems to deliver surveillance testing, diagnostic evaluation, and excisional treatment (if needed). Retrospective data are challenging to obtain and are most likely available when patients have had an ongoing relationship with the system for several years. According to Tiro,^5^ many patients are missing screening history data; therefore, management recommendations are made under conditions of uncertainty when clinicians, at the time of an abnormal result, may not have access to the information needed to assess risk of developing cervical intraepithelial neoplasia 3 or worse (CIN3+). The second essential element—system engagement for surveillance testing—is the focus of the current analysis.

Do clinicians and systems maintain contact with and deliver services to patients to complete recommended surveillance? We addressed this question for patients with positive high-risk (non-16/18 or pooled genotypes) HPV test results and negative for intraepithelial lesion or malignancy (NILM) cytologic findings. This group is of particular concern because US health care systems are shifting to primary HPV screening and cotesting,^6^ making HPV-positive result and NILM cytologic finding a common screening abnormality (range, 6.7%-14.9%).^7,8,9,10^ Furthermore, most studies in the literature to date have monitored colposcopy receipt after a high-grade result^11,12,13,14^; few have studied follow-up after an HPV-positive result and NILM cytologic finding.^15^

Guideline-concordant practice for an HPV-positive result and NILM cytologic finding should include annual surveillance cotesting to monitor for HPV persistence and potential progression of cervical dysplasia.^3^ If findings are negative over 2 subsequent evaluations, then a patient can exit management and return to average-risk screening (ie, cytologic test alone every 3 years; primary HPV or cotesting every 5 years). Evidence supporting these recommendations is based on data from a limited number of settings.^16,17^ To describe how well practice aligns with guidelines, we used longitudinal cohort data from the National Cancer Institute Population-based Research to Optimize the Screening Process (PROSPR) II Cervical Consortium.^6,18^ In 3 diverse health care settings, we aimed to quantify patterns of surveillance testing and associated outcomes for patients after an HPV-positive result and NILM cytologic finding.

## Methods

### Study Setting and Population

The PROSPR II Cervical Consortium evaluates cervical cancer screening processes by contributing data to the METRICS (Multi-level Optimization of the Cervical Cancer Screening Process in Diverse Settings and Populations) cohort.^6,18^ The PROSPR II consortium includes Kaiser Permanente Washington (KPWA), Mass General Brigham (MGB), and Parkland Health (PH). This cohort study was approved by the institutional review boards of KPWA, MGB, and PH, which waived the required informed consent for study participation because research posed minimal risk, did not adversely affect the rights and welfare of participants, and could not practicably be conducted without a waiver. We followed the Strengthening the Reporting of Observational Studies in Epidemiology (STROBE) reporting guideline.

KPWA is a mixed-model, managed care system offering health care and insurance coverage to members in Washington state. MGB is an integrated health care system in Boston, Massachusetts, with 2 hospitals and their affiliated primary care networks. PH is an integrated safety-net system caring for uninsured and underinsured people in Dallas County, Texas. These 3 systems and their respective patient populations have been described previously.^6,18^ In terms of cervical screening practices, the systems differed in adoption of cotesting during the cohort period: 2013 for MGB, 2016 for KPWA, and Papanicolaou test alone was preferred for PH.^6^ The systems had no specific programs to reach out to patients with HPV-positive results and NILM cytologic findings other than documenting recommendation for annual cotesting in the test result report and progress notes. PH allowed for 3-year follow-up after initial HPV-negative result and NILM cytologic finding retest because the 5-year risk of CIN3+ is less than 1%.

### Data Collection

Electronic health record (EHR), administrative data, and state or regional cancer registries were used to identify sociodemographic information, health care use, cytologic and HPV test dates and results, procedure dates and results, pregnancy status, and cancer diagnoses prior to the index result, as reported by Feldman et al.^11^ Screen-eligible patients aged 21 to 65 years entered the analytic cohort at the first qualifying abnormal result (index) from January 2010 to August 2018, with surveillance ascertained through December 2019 (ie, all analytic cohort members had 16 months to receive the first subsequent surveillance test). Qualifying abnormal results included NILM cytologic findings and positive results for a pool of 12 to 14 high-risk HPV genotypes (ie, assay may include 16/18 genotypes in the pool); if the assay specifically tested for HPV-16/18 and findings were positive, we excluded those patients because the guidelines recommend immediate colposcopy.^3^ Race and ethnicity were obtained from documentation in the EHR (Hispanic or Latine; non-Hispanic African American or Black, Asian, White; multiple races and other [including Native Hawaiian or Pacific Islander, Native American or Alaska Native, other race, multiple non-Hispanic races]; and unknown) and were assessed in this study to examine variation in completion of recommended surveillance.

### Statistical Analysis

We assessed all cervical tests and procedures (cytologic test, HPV test, colposcopy, loop electrosurgical excision, cone biopsy, hysterectomy) occurring 16 months after the index HPV-positive result and NILM cytologic finding (hereafter round 1 surveillance). Because ASCCP guidelines recommend annual surveillance, we noted if tests or procedures were received before month 11 (ie, early), and we allowed testing up to month 16 (ie, buffer or grace period).^19^ At the end of round 1 surveillance, we classified cohort members as tested (whether HPV-negative result and NILM cytologic finding or abnormal result), untested (ie, no follow-up), or exited the cohort. Reasons for exit included death, disenrollment from the health care system (KPWA only), moving out of the SEER (Surveillance, Epidemiology, and End Results Program) Seattle or Puget Sound area (KPWA only), moving out of Dallas (PH only). Only patients with an HPV-negative result and NILM cytologic finding in round 1 surveillance were eligible to advance to round 2 surveillance, which had a similar 16-month observation window. An additional reason to exit the cohort in round 2 was reaching the end of the study period (December 31, 2019). Transitions over 2 surveillance rounds from 2010 to 2019 for the total cohort and by health care system were documented. Among patients with no follow-up in round 1, we noted the test status and median (IQR) time to the next event during the cohort period.

To understand factors associated with being untested during round 1 surveillance, we examined the distributions of covariates by test status, overall and by health care system. We also fit multivariate logistic regression models to estimate adjusted odds ratios (AORs) with 95% confidence limits (CLs) for tested vs untested patients. Selection of reference group was based on the largest category for each covariate. Patients missing 1 or more covariate data were dropped from analyses. Health care system was added as a fixed covariate in the overall model due to differences in covariate distributions and the small number of health care systems. Because many patients did not receive any cytologic or HPV tests or procedures during round 1 surveillance, we described the worst cervical cytologic or pathologic outcome documented in the EHR before cohort exit, stratified by round 1 surveillance test status.

All statistical tests comparing cohort member characteristics at the date of the index result by health care system were 2-tailed, with statistical significance set at *P* < .05. Data processing and analyses were conducted between April 2021 and November 2024 using SAS, version 9.4 (SAS Institute Inc), and R, version 4.0.3 (R Project for Statistical Computing).

## Results

### Sample Characteristics by Health Care System

**Figure 1.**
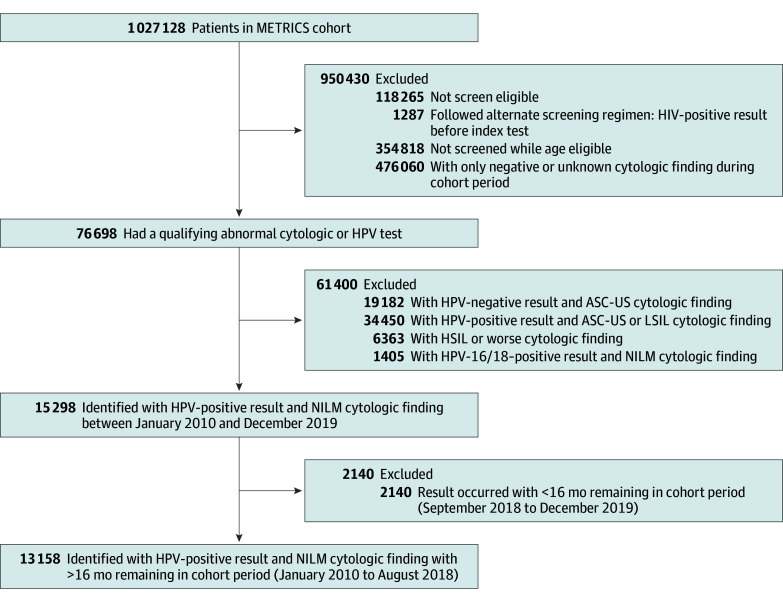
CONSORT Diagram of the PROSPR II METRICS Cohort ASC-US indicates atypical squamous cells of uncertain significance; HPV, human papillomavirus; HSIL, high-grade squamous intraepithelial lesion; LSIL, low-grade squamous intraepithelial lesion; METRICS, Multi-level Optimization of the Cervical Cancer Screening Process in Diverse Settings and Populations; NILM, negative for intraepithelial lesion or malignancy; PROSPR II, Population-based Research to Optimize the Screening Process.

Figure 1 is a flow diagram reporting exclusions and the final sample across 3 health care systems, which included 13 158 patients with an HPV-positive result and NILM cytologic finding. This sample represents 17.2% of all index abnormal results (n = 76 698) and 1.3% of the entire METRICS cohort (n = 1 027 128). All patients were females, of whom 3228 (24.5%) were identified as Hispanic or Latine, 1990 (15.1%) as non-Hispanic African American or Black (hereafter African American or Black), 749 (5.7%) as non-Hispanic Asian (hereafter Asian), and 6559 (49.8%) as non-Hispanic White (hereafter White) individuals, with 404 (3.1%) having other or multiple races and 228 (1.7%) being of unknown race and ethnicity. Sociodemographic characteristics were fairly similar for KPWA and MGB (Table 1); at the index test, most patients at KPWA and MGB were aged 30 to 39 years (1371 [38.4%] and 2820 [42.5%], respectively) and 40 to 49 years (847 [23.7%] and 1636 [24.6%], respectively), of White race (2277 [63.7%] and 4061 [61.2%], respectively), had commercial health insurance (3137 [87.8%] and 4365 [65.7%], respectively), and lived in a Census tract with low to moderate area-level poverty (≤22%). There were more Hispanic or Latine patients at MGB than KPWA (1290 [19.4%] vs 274 [7.7%], respectively), and the systems differed in past screening history, with most patients at MGB having average risk with normal results (3817 [57.5%]), while most KPWA patients had unknown screening history (1840 [51.5%]). The PH patient population was substantially different, of whom most were 40 to 65 years of age (1822 [61.9%]), Hispanic or Latine (1664 [56.5%]), uninsured or covered by government payer programs (2809 [95.4%]), and lived in a Census tract with high area-level poverty (1764 [59.9%]). Across the METRICS cohort sites, most cohort members had a primary care clinician (PCC) of record, were not pregnant, and had at least 1 primary care encounter before the index abnormal result.

**Table 1.** Characteristics of Patients With At Least 1 Qualifying Human Papillomavirus–Positive Result and Negative for Intraepithelial Lesion or Malignancy Cytologic Finding by Health Care System

Characteristic	Participants, No. (%)			
	Total (N = 13 158)	Health care system^a^		
		KPWA (n = 3574)	MGB (n = 6640)	PH (n = 2944)
**Sociodemographic **				
Age group at index result, y				
21-24	412 (3.1)	118 (3.3)	134 (2.0)	160 (5.4)
25-29	735 (5.6)	224 (6.3)	290 (4.4)	221 (7.5)
30-39	4932 (37.5)	1371 (38.4)	2820 (42.5)	741 (25.2)
40-49	3285 (25.0)	847 (23.7)	1636 (24.6)	802 (27.2)
50-65	3794 (28.8)	1014 (28.4)	1760 (26.5)	1020 (34.7)
Race and ethnicity^b^				
African American or Black	1990 (15.1)	263 (7.4)	727 (11.0)	1000 (34.0)
Asian	749 (5.7)	392 (11.0)	313 (4.7)	44 (1.5)
Hispanic or Latine	3228 (24.5)	274 (7.7)	1290 (19.4)	1664 (56.5)
White	6559 (49.8)	2277 (63.7)	4061 (61.2)	221 (7.5)
Multiple races and other	404 (3.1)	242 (6.8)	154 (2.3)	8 (0.3)
Unknown	228 (1.7)	126 (3.5)	95 (1.4)	7 (0.2)
Health insurance or payer in calendar year of index result				
Commercial	7633 (58.0)	3137 (87.8)	4365 (65.7)	131 (4.5)
Medicare	292 (2.2)	41 (1.2)	141 (2.1)	110 (3.7)
Medicaid	2347 (17.8)	101 (2.8)	1899 (28.6)	347 (11.8)
Uninsured, multiple, or other payers^c^	2882 (21.9)	295 (8.3)	235 (3.5)	2352 (79.9)
Unknown	<5 (0.0)	0	0	<5 (0.1)
Area-level poverty at cohort entry, %^d^				
Low: 0-10	5014 (38.1)	1881 (52.6)	2879 (43.4)	254 (8.6)
Moderate: 11-22	4343 (33.0)	1214 (34.0)	2261 (34.1)	868 (29.5)
High: ≥23	3413 (25.9)	391 (10.9)	1258 (19.0)	1764 (59.9)
Missing data	388 (2.9)	88 (2.5)	242 (3.6)	58 (2.0)
**Health care use**				
Has PCC of record in calendar year of index result				
Yes	12 006 (91.2)	3318 (92.8)	6253 (94.2)	2435 (82.7)
No	1152 (8.8)	256 (7.2)	387 (5.8)	509 (17.3)
Time from first system encounter to index result, y^e^				
No prior system encounters	1421 (10.8)	580 (16.2)	490 (7.4)	351 (11.9)
≤1	2173 (16.5)	620 (17.4)	880 (13.3)	673 (22.9)
>1-3	2601 (19.8)	410 (11.5)	1421 (21.4)	770 (26.2)
>3-5	2166 (16.5)	360 (10.1)	1242 (18.7)	564 (19.2)
>5	4797 (36.5)	1604 (44.9)	2607 (39.3)	586 (19.9)
Pregnant at index result				
Yes	351 (2.7)	103 (2.9)	144 (2.2)	104 (3.5)
No	12 807 (97.3)	3471 (97.1)	6496 (97.8)	2840 (96.5)
Past cervical cancer screening at index result^f^				
Under surveillance	1787 (13.6)	198 (5.5)	1099 (16.6)	490 (16.6)
Average risk	6628 (50.4)	1536 (43.0)	3817 (57.5)	1275 (43.3)
Unknown risk	4743 (36.0)	1840 (51.5)	1724 (26.0)	1179 (40.1)
Calendar year of index result				
2010	812 (6.2)	50 (1.4)	323 (4.9)	439 (14.9)
2011	1136 (8.6)	23 (0.6)	442 (6.7)	671 (22.8)
2012	1090 (8.3)	28 (0.8)	627 (9.4)	435 (14.8)
2013	1175 (8.9)	133 (3.7)	804 (12.1)	238 (8.1)
2014	1628 (12.4)	336 (9.4)	1020 (15.4)	272 (9.2)
2015	1671 (12.7)	469 (13.1)	1050 (15.8)	152 (5.2)
2016	2005 (15.2)	801 (22.4)	938 (14.1)	266 (9.0)
2017	2120 (16.1)	974 (27.3)	892 (13.4)	254 (8.6)
2018	1521 (11.6)	760 (21.3)	544 (8.2)	217 (7.4)

Abbreviations: KPWA, Kaiser Permanente Washington; MGB, Mass General Brigham; PCC, primary care clinician; PH, Parkland Health.

^a^ All patient characteristics for each result category were significantly different across the cohort sites (*P* < .001).

^b^ Based on information recorded in the electronic health record using the following mutually exclusive categories in the following order of priority: Hispanic or Latine, regardless of race; single designation of African American or Black, Asian, White; and other, which included Native Hawaiian or Other Pacific Islander, Native American or Alaska Native, other race, and multiple race designations; and unknown, which included cohort members without a documented race or ethnicity.

^c^ Included patients who were not known to have payer coverage during the calendar year of the index abnormal test as well as those using other and/or multiple forms of payers during the calendar year of the index abnormal test. Other insurance or payers included disability program, Ryan White program, family planning program (Title X, Title XX), and the National Breast and Cervical Cancer Early Detection Program; the latter 3 are government-based subsidized programs. This category varied in composition by cohort site: at KPWA, all people in this category had multiple insurance types noted throughout the calendar year; at MGB, 63.4% of people in this category had multiple insurance types, while the remaining 35.7% used other insurance or payers; at PH, 92.0% of people in this category used a government payer, 6.0% had multiple insurance types, and 2.0% were uninsured.

^d^ The 2019 poverty threshold was $13 011 for a 1-person household and $16 521 for a 2-person household. In this study, low-poverty census tracts meant 0% to 10% of households in that tract had a total income below the federal poverty threshold.

^e^ Reflects the time from a patient’s first primary care visit in the health care system to the date of the index abnormal test; this may have occurred prior to the METRICS (Multi-level Optimization of the Cervical Cancer Screening Process in Diverse Settings and Populations) cohort entry. Primary care encounters were documented up to 3 years before METRICS cohort entry.

^f^ Defined as follows: under surveillance, which included people with at least 1 prior abnormal test or diagnostic evaluation or excisional procedure; average risk, which included people with at least 1 prior normal cervical cancer test (eg, human papillomavirus–positive result and negative for intraepithelial lesion or malignancy cytologic finding [NILM] or NILM only) and no prior abnormal tests or cervical procedures; or unknown risk, which included people with no prior test or procedure documented before the index abnormal result.

KPWA had a higher proportion of HPV-positive results and NILM cytologic findings after recommending cotesting in 2016, while MGB had a consistent proportion with HPV-positive results and NILM cytologic findings since 2013. PH exhibited a steady proportion from 2013 onward, likely reflecting the use of cotesting for underscreened and new patients with an abnormal history, which was prevalent in earlier cohort years.

### Transitions Over 2 Rounds of Testing

**Figure 2.**
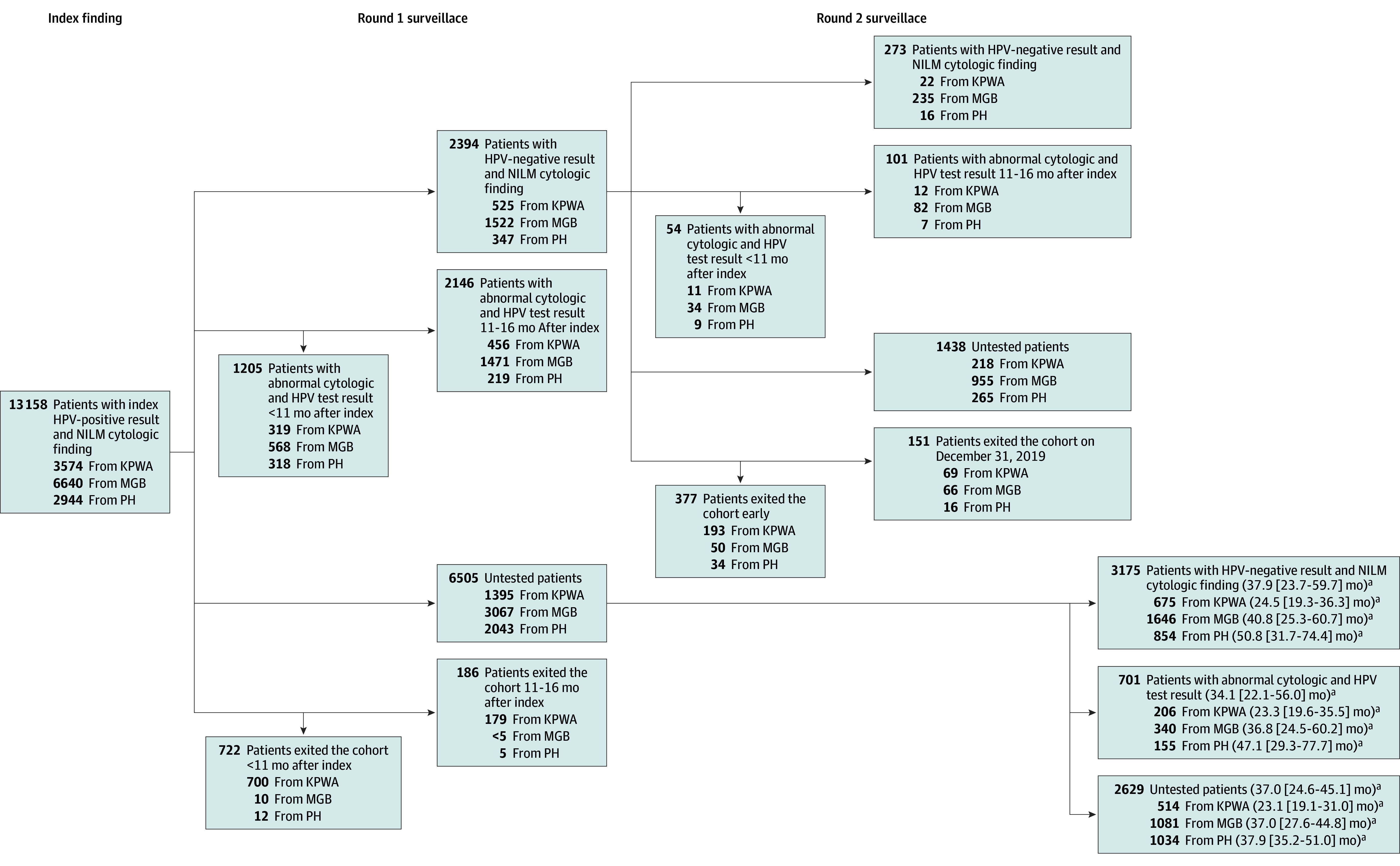
Transitions Over Rounds of Surveillance Testing After Index Human Papillomavirus (HPV)–Positive Result and Negative for Intraepithelial Lesion or Malignancy (NILM) Cytologic Finding, Overall and by Health Care System Round 1 events occurred within 16 months of index result. Round 2 events occurred 22 to 32 months after the index result and within 16 months of the round 1 HPV-negative result and NILM cytologic finding. KPWA indicates Kaiser Permanente Washington; MGB, Mass General Brigham; and PH, Parkland Health. ^a^Median (IQR) time to next event.

By the end of round 1 surveillance (Figure 2), only 43.7% of the 13 158 patients were tested: 18.2% (2394) had a documented HPV-negative result and NILM cytologic finding, and 25.5% (3351) had an abnormal result (ie, 58.3% of the 5745 tested had persistent HPV or progressed to dysplasia). During round 1 surveillance, many patients were untested despite remaining enrolled (overall: 49.4% [6505]; across sites: 39.0% [1395] to 69.4% [2043]), and fewer patients exited the cohort without being tested (overall: 6.9% [908]; across sites: 0.2% [12] to 24.6% [879]). Variation across sites is shown in Figure 2. Among the 2394 eligible for round 2 surveillance, only 11.4% (273) had a documented second HPV-negative result and NILM cytologic finding (ie, 2.1% of the 13 158 total cohort); 6.5% (155) had an abnormal result early or during round 2 surveillance, 60.1% (1438) were untested (41.5%-76.4% across sites) and 22.1% (528) exited before the end of the 16-month window.

Investigation of each health care system showed substantially different patterns (Figure 2). At KPWA, 24.6% of patients exited analysis before the end of round 1 surveillance; almost all disenrolled from the health plan. At PH, 69.4% were untested during round 1 surveillance. MGB had the highest proportion of patients with an HPV-negative result and NILM cytologic finding during round 1 (22.9%) and round 2 (15.4%) surveillance; MGB lost 46.2% during round 1 surveillance due to lack of testing.

### Factors Associated With Round 1 Testing

Table 2 reports patient characteristics associated with testing during round 1 surveillance. Overall, groups less likely to have timely testing were younger adults (aged 25-29 vs 30-39 years: AOR, 0.65; 95% CL, 0.53-0.81), African American or Black patients (compared with White patients: AOR, 0.78; 95% CL, 0.68-0.89), and those with Medicaid (vs commercial insurance: AOR, 0.81; 95% CL, 0.72-0.91); patients with a PCC were more likely to have timely testing (AOR, 1.44; 95% CL, 1.21-1.70). Patients under surveillance or with average risk (vs unknown risk or no prior screen) were more likely to be tested (Table 2). KPWA and PH had significantly lower timely surveillance testing compared with MGB (AOR, 0.63 [95% CL, 0.57-0.71] and 0.32 [95% CL, 0.26-0.38]). Within the health care systems, there were some unique patterns of association. At KPWA, timely testing was lower among those aged 50 to 65 years compared with those aged 30 to 39 years (296 of 756 [39.2%] vs 337 of 769 [43.8%]) and higher among those who had no prior encounters and whose first encounter was 1 to 3 years compared with greater than 5 years before index (129 of 292 [44.2%] and 104 of 229 [45.4%] vs 479 of 1174 [40.8%]). At MGB, testing was lower among the uninsured patients and those with Medicaid vs commercial insurance (80 of 193 [41.5%] and 753 of 1677 [44.9%] vs 1958 of 3764 [52.0%]). At PH, Asian patients were more likely to be tested (13 of 39 [33.3%]). While not significant, the direction of association for African American or Black compared with White patients was the same across all sites.

**Table 2.** Association Between Patient Characteristics and Surveillance Testing During Round After an Index Human Papillomavirus–Positive Result and Negative for Intraepithelial Lesion or Malignancy Cytologic Finding

Characteristic	Participants, No. (%)											
	Total			Health care system								
				KPWA			MGB			PH		
	Tested (n = 4312)	Untested (n = 6241)	AOR (95% CL)^a^	Tested (n = 916)	Untested (n = 1332)	AOR (95% CL)^a^	Tested (n = 2851)	Untested (n = 2904)	AOR (95% CL)^a^	Tested (n = 545)	Untested (n = 2005)	AOR (95% CL)^a^
**Sociodemographic**												
Age group at index result, y												
21-24	85 (2.0)	175 (2.8)	0.89 (0.67-1.18)	15 (1.6)	36 (2.7)	0.56 (0.29-1.08)	52 (1.8)	58 (2.0)	1.07 (0.72-1.58)	18 (3.3)	81 (4.0)	1.07 (0.61-1.90)
25-29	147 (3.4)	354 (5.7)	0.65 (0.53-0.81)	39 (4.3)	63 (4.7)	0.82 (0.53-1.26)	90 (3.2)	158 (5.4)	0.65 (0.49-0.86)	18 (3.3)	133 (6.6)	0.55 (0.32-0.95)
30-39	1606 (37.2)	2119 (34.0)	1 [Reference]	337 (36.8)	432 (32.4)	1 [Reference]	1137 (39.9)	1204 (41.5)	1 [Reference]	132 (24.2)	483 (24.1)	1 [Reference]
40-49	1151 (26.7)	1626 (26.1)	1.01 (0.91-1.12)	229 (25.0)	341 (25.6)	0.84 (0.67-1.06)	758 (26.6)	727 (25.0)	1.03 (0.90-1.17)	164 (30.1)	558 (27.8)	1.17 (0.89-1.53)
50-65	1323 (30.7)	1967 (31.5)	0.97 (0.88-1.08)	296 (32.3)	460 (34.5)	0.77 (0.62-0.96)	814 (28.6)	757 (26.1)	1.02 (0.89-1.17)	213 (39.1)	750 (37.4)	1.16 (0.89-1.51)
Race and ethnicity^b^												
African American or Black	499 (11.6)	1188 (19.0)	0.78 (0.68-0.89)	59 (6.4)	110 (8.3)	0.75 (0.53-1.06)	286 (10.0)	342 (11.8)	0.86 (0.72-1.03)	154 (28.3)	736 (36.7)	0.86 (0.57-1.29)
Asian	250 (5.8)	319 (5.1)	0.97 (0.81-1.16)	107 (11.7)	153 (11.5)	0.92 (0.70-1.22)	130 (4.6)	140 (4.8)	0.91 (0.71-1.17)	13 (2.4)	26 (1.3)	2.23 (1.02-4.86)
Hispanic or Latine	957 (22.2)	1812 (29)	0.96 (0.85-1.09)	74 (8.1)	114 (8.6)	0.86 (0.62-1.17)	543 (19.1)	617 (21.3)	0.96 (0.82-1.13)	340 (62.4)	1081 (53.9)	1.12 (0.76-1.66)
White	2481 (57.5)	2746 (44.0)	1 [Reference]	612 (66.8)	854 (64.1)	1 [Reference]	1831 (64.2)	1738 (59.9)	1 [Reference]	38 (7.0)	154 (7.7)	1 [Reference]
Multiple races or other	125 (2.9)	176 (2.8)	0.92 (0.72-1.17)	64 (7.0)	101 (7.6)	0.88 (0.62-1.23)	61 (2.1)	67 (2.3)	1.08 (0.75-1.55)	0	8 (0.4)	NA
Insurance or payer in calendar year of index result												
Commercial	2782 (64.5)	3089 (49.5)	1 [Reference]	804 (87.8)	1186 (89)	1 [Reference]	1958 (68.7)	1806 (62.2)	1 [Reference]	20 (3.7)	97 (4.8)	1 [Reference]
Medicare	88 (2.0)	163 (2.6)	0.89 (0.67-1.19)	9 (1.0)	21 (1.6)	0.68 (0.30-1.51)	60 (2.1)	61 (2.1)	0.92 (0.64-1.34)	19 (3.5)	81 (4.0)	1.20 (0.59-2.46)
Medicaid	830 (19.3)	1199 (19.2)	0.81 (0.72-0.91)	28 (3.1)	41 (3.1)	1.05 (0.64-1.73)	753 (26.4)	924 (31.8)	0.79 (0.69-0.90)	49 (9.0)	234 (11.7)	1.06 (0.59-1.91)
Uninsured, multiple, or other^c^	612 (14.2)	1790 (28.7)	0.99 (0.83-1.18)	75 (8.2)	84 (6.3)	1.38 (0.98-1.93)	80 (2.8)	113 (3.9)	0.69 (0.51-0.94)	457 (83.9)	1593 (79.5)	1.31 (0.79-2.17)
Area-level poverty at cohort entry, %^d^												
Low: 0-10	1890 (43.8)	2181 (35.0)	1 [Reference]	511 (55.8)	738 (55.4)	1 [Reference]	1333 (46.8)	1266 (43.6)	1 [Reference]	46 (8.4)	177 (8.8)	1 [Reference]
Moderate: 11-22	1454 (33.7)	2070 (33.2)	0.97 (0.88-1.07)	298 (32.5)	447 (33.6)	0.96 (0.80-1.16)	992 (34.8)	1031 (35.5)	0.99 (0.88-1.12)	164 (30.1)	592 (29.5)	1.04 (0.71-1.53)
High: ≥23	968 (22.5)	1990 (31.9)	0.96 (0.85-1.08)	107 (11.7)	147 (11.0)	1.06 (0.80-1.41)	526 (18.5)	607 (20.9)	0.93 (0.80-1.09)	335 (61.5)	1236 (61.7)	1.04 (0.72-1.50)
**Health care use**												
Has PCC of record in calendar year of index result												
Yes	4095 (95.0)	5651 (90.6)	1.44 (1.21-1.70)	871 (95.1)	1252 (94.0)	1.08 (0.71-1.64)	2754 (96.6)	2681 (92.3)	2.14 (1.67-2.75)	470 (86.2)	1718 (85.7)	1.01 (0.74-1.39)
No	217 (5.0)	590 (9.5)	1 [Reference]	45 (4.9)	80 (6.0)	1 [Reference]	97 (3.4)	223 (7.7)	1 [Reference]	75 (13.8)	287 (14.3)	1 [Reference]
Time from first system encounter to index result, y^e^												
No prior system encounters	348 (8.1)	658 (10.5)	1.05 (0.88-1.27)	129 (14.1)	163 (12.2)	1.71 (1.19-2.45)	173 (6.1)	240 (8.3)	0.77 (0.59-1.00)	46 (8.4)	255 (12.7)	1.05 (0.66-1.66)
≤1	570 (13.2)	1072 (17.2)	1.03 (0.88-1.21)	128 (14.0)	202 (15.2)	1.34 (0.95-1.89)	323 (11.3)	397 (13.7)	0.83 (0.67-1.03)	119 (21.8)	473 (23.6)	1.39 (0.95-2.04)
>1-3	824 (19.1)	1248 (20.0)	1.01 (0.88-1.15)	104 (11.4)	125 (9.4)	1.59 (1.13-2.23)	583 (20.5)	628 (21.6)	0.83 (0.71-0.98)	137 (25.1)	495 (24.7)	1.06 (0.76-1.49)
>3-5	691 (16.0)	1122 (18.0)	0.85 (0.75-0.96)	76 (8.3)	147 (11.0)	0.77 (0.56-1.05)	503 (17.6)	585 (20.1)	0.76 (0.65-0.88)	112 (20.6)	390 (19.5)	1.10 (0.79-1.54)
>5	1879 (43.6)	2141 (34.3)	1 [Reference]	479 (52.3)	695 (52.2)	1 [Reference]	1269 (44.5)	1054 (36.3)	1 [Reference]	131 (24.0)	392 (19.6)	1 [Reference]
Cervical cancer screening history at index result^f^												
Under surveillance	591 (13.7)	810 (13.0)	1.34 (1.16-1.56)	50 (5.5)	71 (5.3)	1.64 (1.05-2.57)	444 (15.6)	496 (17.1)	1.06 (0.88-1.28)	97 (17.8)	243 (12.1)	2.36 (1.68-3.31)
Average risk	2542 (59.0)	3110 (49.8)	1.47 (1.30-1.66)	489 (53.4)	656 (49.3)	1.82 (1.38-2.41)	1771 (62.1)	1588 (54.7)	1.28 (1.09-1.51)	282 (51.7)	866 (43.2)	1.90 (1.45-2.49)
Unknown risk	1179 (27.3)	2321 (37.2)	1 [Reference]	377 (41.2)	605 (45.4)	1 [Reference]	636 (22.3)	820 (28.2)	1 [Reference]	166 (30.5)	896 (44.7)	1 [Reference]
Health care system												
KPWA	916 (21.2)	1332 (21.3)	0.63 (0.57-0.71)	NA	NA	NA	NA	NA	NA	NA	NA	NA
MGB	2851 (66.1)	2904 (46.5)	1 [Reference]	NA	NA	NA	NA	NA	NA	NA	NA	NA
PH	545 (12.6)	2005 (32.1)	0.32 (0.26-0.38)	NA	NA	NA	NA	NA	NA	NA	NA	NA

Abbreviations: AOR, adjusted odds ratio; CL, confidence limit; KPWA, Kaiser Permanente Washington; MGB, Mass General Brigham; NA, not applicable; PCC, primary care clinician; PH, Parkland Health.

^a^ In addition to the patient characteristics and health care system listed in the table, the multivariate logistic regression models adjusted for calendar year of the index abnormal result.

^b^ Based on information recorded in the electronic health record using the following mutually exclusive categories in the following order of priority: Hispanic or Latine, regardless of race; single designation of African American or Black, Asian; other, which included Native Hawaiian or Other Pacific Islander, Native American or Alaska Native, other race, and multiple non-Hispanic race designations; and unknown, which included cohort members without a documented race or ethnicity.

^c^ Included patients who were not known to have payer coverage during the calendar year of the index abnormal test as well as patients using other and/or multiple forms of payers during the calendar year of the index abnormal test. Other insurance or payers included disability program, Ryan White program, family planning program (Title X, Title XX), and the National Breast and Cervical Cancer Early Detection Program; the latter 3 are government-based subsidized programs. This category varied in composition by cohort site: at KPWA, all people in this category had multiple insurance types noted throughout the calendar year; at MGB, 63.4% of people in this category had multiple insurance types, while the remaining 35.7% used other insurance or payers; at PH, 92.0% of people in this category used a government payer, 6.0% had multiple insurance types, and 2.0% were uninsured.

^d^ The 2019 poverty threshold was $13 011 for a 1-person household and $16 521 for a 2-person household. In this study, low-poverty census tracts meant 0% to 10% of households in that tract had a total income below the federal poverty threshold.

^e^ Reflects the time from a patient’s first primary care visit in the health care system to the date of the index abnormal test; this may have occurred prior to the METRICS (Multi-level Optimization of the Cervical Cancer Screening Process in Diverse Settings and Populations) cohort entry. Primary care encounters were documented up to 3 years before METRICS cohort entry.

^f^ Defined as follows: under surveillance, which included people with at least 1 prior abnormal test or diagnostic evaluation or excisional procedure; average risk, which included people with at least 1 prior normal cervical cancer test (eg, human papillomavirus–positive result and negative for intraepithelial lesion or malignancy cytologic finding [NILM] or NILM only) and no prior abnormal tests or cervical procedures; or unknown risk, which included people with no prior test or procedure documented before the index abnormal result.

### Worst Outcome by Round 1 Test Status

Table 3 shows the worst outcome by the end of the cohort period among untested patients (n = 6505) and among those receiving an HPV-negative result and NILM cytologic finding (n = 2394) during round 1 surveillance. Across systems, the proportion of patients initially untested in round 1 surveillance, who remained untested through the end of the cohort period, was 40.4% (2629); across sites, PH had the highest proportion untested (50.6% [1034]). For those who had subsequent pathology, there were 10 cancers (0.2%) and 54 in situ cancers (0.8%) ultimately detected among those untested in round 1 surveillance. The worst outcome distribution was similar across systems with a few exceptions. More cohort members at MGB were diagnosed with cancer (5 [0.2%]), carcinoma in situ or adenocarcinoma in situ (32 [1.0%]), or high-grade squamous intraepithelial lesion (87 [2.8%]). The proportion of untested patients with HPV-negative results and NILM cytologic findings at cohort end was similar across health care systems (34.1%-39.0%). Among the 2394 patients who received an HPV-negative result and NILM cytologic finding during round 1 surveillance, the worst outcome distribution observed by the end of the cohort period was 5 (0.2%) for carcinoma in situ or adenocarcinoma in situ, 16 (0.7%) for high-grade squamous intraepithelial lesion, 89 (3.7%) for atypical squamous cells of uncertain significance or low-grade squamous intraepithelial lesion, 6 (0.3%) for HPV-16/18–positive results and NILM cytologic findings, and 132 (5.5%) for HPV-positive (pooled or other genotype) results and NILM cytologic findings.

**Table 3.** Worst Cytologic or Pathologic Outcome by End of the Cohort Period for Untested Patients or Patients With a HPV-Negative Result and NILM Cytologic Findings in Round 1, Overall and by Health Care System

Round 1 outcome	Participants, No. (%)							
	Total		Health care system					
			KPWA		MGB		PH	
	Untested (n = 6505)	HPV-negative and NILM finding (n = 2394)	Untested (n = 1395)	HPV-negative and NILM finding (n = 525)	Untested (n = 3067)	HPV- negative and NILM finding (n = 1522)	Untested (n = 2043)	HPV- negative and NILM finding (n = 347)
No additional cytologic or pathologic test^a^^,^^b^	2629 (40.4)	1259 (52.6)	514 (36.9)	422 (80.4)	1081 (35.3)	652 (42.8)	1034 (50.6)	185 (53.3)
≥1 Subsequent cytologic or pathologic test, worst diagnosis outcome^c^^,^^d^	3876 (59.6)	1135 (47.4)	881 (63.2)	103 (19.6)	1986 (64.8)	870 (57.2)	1009 (49.4)	162 (46.7)
Cancer	10 (0.2)	0	<5 (<0.5)	0	5 (0.2)	0	<5 (<0.5)	0
CIS or AIS	54 (0.8)	5 (0.2)	<5 (<0.5)	0	32 (1.0)	0	18 (1.8)	0
HSIL	136 (2.1)	16 (0.7)	23 (1.6)	0	87 (2.8)	14 (0.9)	26 (1.3)	<5 (<0.5)
ASC-US or LSIL	594 (9.1)	89 (3.7)	142 (10.2)	11 (2.1)	299 (9.7)	66 (4.3)	153 (7.5)	12 (3.5)
HPV-16/18 genotypes positive and NILM	51 (0.8)	6 (0.3)	11 (0.8)	0	40 (1.3)	6 (0.4)	0	0
HPV (pooled or other genotype) positive and NILM	635 (9.8)	132 (5.5)	147 (10.5)	9 (1.7)	390 (12.7)	114 (7.5)	98 (4.8)	9 (2.6)
HPV negative and NILM	2361 (36.3)	873 (36.5)	544 (39.0)	77 (14.7)	1121 (36.6)	659 (43.3)	696 (34.1)	137 (39.5)
Insufficient or missing outcome	35 (0.5)	14 (0.6)	9 (0.6)	6 (1.1)	12 (0.4)	6 (0.4)	14 (0.7)	<5 (<0.5)

Abbreviations: AIS, adenocarcinoma in situ; ASC-US, atypical squamous cells of uncertain significance; CIS, carcinoma in situ; HPV, human papillomavirus; HSIL, high-grade squamous intraepithelial lesion; KPWA, Kaiser Permanente Washington; LSIL, low-grade squamous intraepithelial lesion; MGB, Mass General Brigham; NILM, negative for intraepithelial lesion or malignancy; PH, Parkland Health.

^a^ For untested patients (no follow-up) during round 1 and additional cytologic or pathologic test, the median (IQR) time from the index HPV-positive result and NILM cytologic finding to cohort exit was 37.0 (24.6-45.1) months overall, 23.1 (19.1-31.0) months for KPWA, 37.0 (27.6-44.8) months for MGB, and 37.9 (35.2-51.0) months for PH.

^b^ For patients with an HPV-negative result and NILM cytologic finding during round 1 and no additional cytologic or pathologic test, the median (IQR) time from index HPV-positive result and NILM cytologic finding to cohort exit was 33.3 (22.8-48.9) months overall, 25.4 (20.3-36.1) months for KPWA, 38.2 (25.0-52.2) months for MGB, and 40.8 (28.5-59.0) months for PH.

^c^ For untested patients (no follow-up) during round 1 and at least 1 subsequent cytologic or pathologic test, the median (IQR) time from index HPV-positive result and NILM cytologic finding to cohort exit was 62.3 (42.1-84.7) months overall for those with a subsequent cytologic or pathologic test, 38.6 (27.9-51.9) months for KPWA, 64.4 (47.3-82.1) months for MGB, and 88.2 (64.6-101.3) months for PH. The median (IQR) time from index HPV-positive result and NILM cytologic finding to next cytologic or pathologic test was 31.8 (21.2-51.3) months overall, 22.4 (18.6-34.0) months for KPWA, 32.7 (21.8-52.3) months for MGB, and 42.9 (27.4-67.9) months for PH.

^d^ For those with an HPV-negative result and NILM cytologic finding in round 1 and at least 1 subsequent cytologic or pathologic test, the median (IQR) time from index HPV-positive result and NILM cytologic finding to cohort exit was 71.8 (55.4-93.3) months overall for those with a subsequent cytologic or pathologic test, 47.8 (33.8-61.2) months for KPWA, 71.4 (56.8-90.3) months for MGB, and 94.8 (74.3-105.1) months for PH. The median (IQR) time from index HPV-positive result and NILM cytologic finding to next cytologic or pathologic test was 48.6 (33.3-63.4) months overall, 33.6 (26.0-47.9) months for KPWA, 48.9 (35.0-63.6) months for MGB, and 55.3 (39.1-75.3) months for PH.

## Discussion

In the METRICS cohort from 2010 to 2018, 17.2% of patients with index abnormal tests had high-risk, non-16/18 genotype HPV-positive results with NILM cytologic findings, and 43.7% of patients received an initial surveillance test during the recommended time frame, although an additional 6.9% exited the cohort before the end of the recommended surveillance window. Of those tested in round 1 surveillance, 58.3% (3351 of 5745) showed HPV persistence or progression toward dysplasia, while the remainder had no HPV infection or progression. Groups who were less likely to have timely testing were younger adults (aged 25-29 years), African American or Black patients, and those with Medicaid, while retesting was higher among patients with a PCC and who had prior screening. When reviewing 2 surveillance rounds, only 2.1% (273 of 13 158) could be identified as eligible to exit surveillance.

We observed substantial variation across the 3 health care systems. The distribution of HPV-positive results and NILM cytologic findings across calendar years reflects differences in adoption of cotesting.^6^ The proportion tested varied substantially, with KPWA unable to track surveillance receipt for 25.5% due to patient disenrollment from the health plan, and PH showing 69.4% untested rate after 16 months, with no indication that patients exited the system. Collectively, the data showed low-level adherence to the annual surveillance regimen recommended by ASCCP guidelines and suggest that abnormal HPV-positive results and NILM cytologic findings may be challenging for health care systems to track and follow to resolution. This challenge may have serious consequences given that these patients have a 0.55% to 3.99% 5-year risk of developing CIN3 or worse.^16^

Our findings are similar to those of the New Mexico HPV Pap Registry^19^ and other countries with organized cancer screening programs (eg, England and Australia),^20,21^ with differences that are potentially attributable to evaluation design. The other studies^20,21^ reported that 29% to 36% of patients were retested. New Mexico allowed an 18-month grace period. Australian participants received these results at different times during the 2-year observation period; thus, the requisite retesting time of 12 months had not yet elapsed for many participants (ie, underestimate of program delivery).^21^ Analysis of the English program^20^ showed the proportion of patients retesting increased from 36% at 1 year to 45% at 2 years. Longer time frames were not assessed as the English program invited all participants for rescreening every 3 years. In New Mexico, approximately 51% of patients were untested after 18 months.^19^ In terms of outcomes, Smith et al^21^ found that 60.9% of participants continued to have other high-risk HPV-positive results at retest and were subsequently recommended to receive a colposcopy. The New Mexico registry observed an increase in biopsies at 12 months after an index HPV-positive result and NILM cytologic finding, consistent with performing colposcopy for persistently abnormal result. None of the aforementioned programs can comment on whether patients were infected with a different HPV type at each visit because all programs genotype only for HPV-16 and 18, reflecting the reality of most screening programs.

To effectively balance screening harms and benefits, clinical teams must be able to monitor delivery of annual surveillance cotests and, if necessary, conduct outreach. Qualitative data collected from the METRICS cohort sites reveal that EHRs are not built to easily document prior screening history, support tracking of patients under surveillance (eg, patient care registry functionality^22,23^), or alert clinicians when patients are overdue for an annual surveillance cotest (unpublished data from interviews with clinicians and staff; personal email communication with R. Higashi, PhD, February 2024). Analysis of the METRICS cohort suggests implementation of effective interventions similar to those in the studies by Atlas et al^24^ and Paskett et al^25^ are needed to track, remind, and engage patients with this particular abnormal test result.

Our data suggest there may be important health consequences for those not receiving annual surveillance. There were 10 invasive cancers detected in patients untested in round 1 surveillance. Our analysis may underestimate the impact of lack of surveillance testing given that 40.4% of untested patients in round 1 surveillance had no subsequent cytologic or pathologic results documented in their EHRs by the end of the cohort period. Underestimation may be even higher for the safety-net system (PH), wherein 50.6% of patients were untested by the end.

### Study Implications

The findings highlight several factors that are challenging for analyses of cervical cancer surveillance in the US. First, health care delivery is fragmented.^26^ We observed approximately 25% of cohort members disenrolled in 1 system. Churn in health insurance plays a role in the movement between health care systems, making access to and tracking of consistent care difficult.^27^ Second, limited transportability of records across systems^28,29,30^ and inconsistent availability of health information exchanges across regions^31,32^ rendered it difficult to fully identify who received timely care after an abnormal result. It is within this context that we observed low incidence of annual surveillance for patients with HPV-positive results and NILM cytologic findings, as recommended by ASCCP management guidelines. Care delivery is further complicated when applying guidelines under conditions of uncertainty; 38% of individuals had unknown screening history prior to their index abnormal result.^5^ Cervical outcome data over longer follow-up periods are needed to elucidate the consequences of low adherence to guidelines and barriers to surveillance, especially because fragmentation may increase cervical cancer disparities.

### Limitations

This study has several limitations. First, data represented 3 geographically distinct health care systems and are not representative of the US or US regions. Furthermore, people who disenrolled (due to change in employer or insurance contract) or left a health care system (and did not notify their clinician) may have received follow-up elsewhere; thus, data capture may be incomplete and may underestimate surveillance. Regions with robust health information exchanges should explore the likelihood of receiving timely surveillance at another health care system and expand measurement beyond population-based screening.^33^ Second, the index HPV-positive result and NILM cytologic finding may not have been a patient’s first sign of HPV infection. In the analytic cohort, 13.6% of patients were under surveillance (ie, had a prior abnormal cytologic finding, positive HPV test result, or pathology result). Thus, a clinician may have recommended immediate colposcopy instead of annual surveillance. We observed that 9.2% of patients received tests or procedures less than 11 months after the index result, suggesting some had more intensive follow-up. There were 453 patients aged 21 to 24 years with an HPV-positive result and NILM cytologic finding, which is discordant with screening guidelines. Third, some of our analyses may not be germane to health care systems that modify elements of the ASCCP guidelines (eg, PH allows for 3-year follow-up after an initial HPV-negative result and NILM cytologic finding retest because the 5-year risk of CIN3+ is <1%).

## Conclusions 

In this cohort study of patients with HPV-positive results and NILM cytologic findings, less than half of the cohort received an initial surveillance cotest at 1 year, with substantial variation across the 3 participating health care systems. Future research should evaluate if the following patient groups have nontimely surveillance after HPV-positive results and NILM cytologic findings: younger (aged 25-29 years), African American or Black, and with Medicaid coverage individuals. Health care systems should monitor the delivery of annual surveillance and gather evidence on interventions to optimize delivery to patient groups at risk for low-level adherence, such as those experiencing adverse social determinants of health. The findings suggest substantial system-level barriers to implementing management guidelines. Unintended, poor cancer outcomes may result.

## Notes

Supplement.
Data Sharing Statement

## References

[1] Alonso-Coello P, Oxman AD, Moberg J, ; GRADE Working Group. GRADE Evidence to Decision (EtD) frameworks: a systematic and transparent approach to making well informed healthcare choices. 2: Clinical practice guidelines. BMJ. 2016;353:i2089. doi:10.1136/bmj.i2089 27365494

[2] Alonso-Coello P, Schünemann HJ, Moberg J, ; GRADE Working Group. GRADE Evidence to Decision (EtD) frameworks: a systematic and transparent approach to making well informed healthcare choices. 1: Introduction. BMJ. 2016;353:i2016. doi:10.1136/bmj.i2016 27353417

[3] Perkins RB, Guido RS, Castle PE, ; 2019 ASCCP Risk-Based Management Consensus Guidelines Committee. 2019 ASCCP risk-based management consensus guidelines for abnormal cervical cancer screening tests and cancer precursors. J Low Genit Tract Dis. 2020;24(2):102-131. doi:10.1097/LGT.0000000000000525 32243307 PMC7147428

[4] Perkins RB, Fuzzell LN, Lake P, . Incorporating stakeholder feedback in guidelines development for the management of abnormal cervical cancer screening tests. J Low Genit Tract Dis. 2020;24(2):167-177. doi:10.1097/LGT.0000000000000524 32243312 PMC7147423

[5] Tiro JA. Reflections on the shift from average to risk-based cancer screening. Paper presented at: 48th Annual Meeting of the American Society of Preventive Oncology; March 18, 2024; Chicago, Illinois.

[6] Haas JS, Cheng D, Yu L, . Variation in the receipt of human papilloma virus co-testing for cervical screening: individual, provider, facility and healthcare system characteristics. Prev Med. 2022;154:106871. doi:10.1016/j.ypmed.2021.106871 34762966 PMC8724456

[7] Wright TC Jr, Stoler MH, Sharma A, Zhang G, Behrens C, Wright TL; ATHENA (Addressing THE Need for Advanced HPV Diagnostics) Study Group. Evaluation of HPV-16 and HPV-18 genotyping for the triage of women with high-risk HPV+ cytology-negative results. Am J Clin Pathol. 2011;136(4):578-586. doi:10.1309/AJCPTUS5EXAS6DKZ 21917680

[8] Tao X, Zhang H, Li J, . Prevalence of HPV-16/18 genotypes and immediate histopathologic correlation results in a Chinese population with negative cytology and positive high-risk HPV testing. Cancer Cytopathol. 2019;127(10):650-657. doi:10.1002/cncy.22180 31532582

[9] Song F, Du H, Xiao A, . Type-specific distribution of cervical hrHPV infection and the association with cytological and histological results in a large population-based cervical cancer screening program: baseline and 3-year longitudinal data. J Cancer. 2020;11(20):6157-6167. doi:10.7150/jca.48357 32922555 PMC7477419

[10] Safaeian M, Schiffman M, Gage J, Solomon D, Wheeler CM, Castle PE. Detection of precancerous cervical lesions is differential by human papillomavirus type. Cancer Res. 2009;69(8):3262-3266. doi:10.1158/0008-5472.CAN-08-4192 19351830 PMC3155840

[11] Feldman S, Lykken JM, Haas JS, . Factors associated with timely colposcopy following an abnormal cervical cancer test result. Prev Med. 2022;164:107307. doi:10.1016/j.ypmed.2022.107307 36270434 PMC9808794

[12] Tosteson AN, Beaber EF, Tiro J, ; PROSPR consortium. Variation in screening abnormality rates and follow-up of breast, cervical and colorectal cancer screening within the PROSPR consortium. J Gen Intern Med. 2016;31(4):372-379. doi:10.1007/s11606-015-3552-7 26658934 PMC4803707

[13] Barlow WE, Beaber EF, Geller BM, . Evaluating screening participation, follow-up, and outcomes for breast, cervical, and colorectal cancer in the PROSPR consortium. J Natl Cancer Inst. 2020;112(3):238-246. doi:10.1093/jnci/djz137 31292633 PMC7073922

[14] Raman SR, Brown JS, Curtis LH, . Cancer screening results and follow-up using routinely collected electronic health data: estimates for breast, colon, and cervical cancer screenings. J Gen Intern Med. 2019;34(3):341-343. doi:10.1007/s11606-018-4697-y 30350029 PMC6420541

[15] Wu M, Ma X, Li H, . Which is the best management for women with normal cervical cytologic findings despite positivity for non-16/18 high risk human papillomaviruses? Front Public Health. 2022;10:950610. doi:10.3389/fpubh.2022.950610 36438260 PMC9682294

[16] Egemen D, Cheung LC, Chen X, . Risk estimates supporting the 2019 ASCCP risk-based management consensus guidelines. J Low Genit Tract Dis. 2020;24(2):132-143. doi:10.1097/LGT.0000000000000529 32243308 PMC7147417

[17] Saraiya M, Cheung LC, Soman A, . Risk of cervical precancer and cancer among uninsured and underserved women from 2009 to 2017. Am J Obstet Gynecol. 2021;224(4):366.e1-366.e32. doi:10.1016/j.ajog.2020.10.001 33035473 PMC8009811

[18] Kamineni A, Tiro JA, Beaber EF, ; PROSPR consortium. Cervical cancer screening research in the PROSPR I consortium: rationale, methods and baseline findings from a US cohort. Int J Cancer. 2019;144(6):1460-1473. doi:10.1002/ijc.31940 30353911 PMC6941787

[19] Perkins RB, Adcock R, Benard V, ; New Mexico HPV Pap Registry (NMHPVPR) Steering Committee. Clinical follow-up practices after cervical cancer screening by co-testing: a population-based study of adherence to U.S. guideline recommendations. Prev Med. 2021;153:106770. doi:10.1016/j.ypmed.2021.106770 34416221 PMC8595756

[20] Rebolj M, Cuschieri K, Mathews CS, Pesola F, Denton K, Kitchener H; HPV Pilot Steering Group. Extension of cervical screening intervals with primary human papillomavirus testing: observational study of English screening pilot data. BMJ. 2022;377:e068776. doi:10.1136/bmj-2021-068776 35640960 PMC9153243

[21] Smith MA, Sherrah M, Sultana F, . National experience in the first two years of primary human papillomavirus (HPV) cervical screening in an HPV vaccinated population in Australia: observational study. BMJ. 2022;376:e068582. doi:10.1136/bmj-2021-068582 35354610 PMC8965648

[22] Miller RS, Mitchell K, Myslinski R, Rising J. Chapter 1: health information technology (IT) and patient registries. In: Gliklich RE, Leavy MB, Dreyer NA, eds. Tools and Technologies for Registry Interoperability, Registries for Evaluating Patient Outcomes: A User's Guide. 3rd Edition, Addendum 2. Agency for Healthcare Research and Quality; 2019. 31891455

[23] Ehrenstein V, Kharrazi H, Lehmann H, Taylor CO. Chapter 4: obtaining data from electronic health records. In: Gliklich RE, Leavy MB, Dreyer NA, . Tools and Technologies for Registry Interoperability, Registries for Evaluating Patient Outcomes: A User's Guide. 3rd Edition, Addendum 2. Agency for Healthcare Research and Quality; 2019. 31891455

[24] Atlas SJ, Tosteson ANA, Wright A, . A multilevel primary care intervention to improve follow-up of overdue abnormal cancer screening test results: a cluster randomized clinical trial. JAMA. 2023;330(14):1348-1358. doi:10.1001/jama.2023.18755 37815566 PMC10565610

[25] Paskett ED, Dudley D, Young GS, ; PNRP Investigators. Impact of patient navigation interventions on timely diagnostic follow up for abnormal cervical screening. J Womens Health (Larchmt). 2016;25(1):15-21. doi:10.1089/jwh.2014.5094 26625131 PMC4741208

[26] Doty MM, Tikkanen R, Shah A, Schneider EC. Primary care physicians’ role in coordinating medical and health-related social needs in eleven countries. Health Aff (Millwood). 2020;39(1):115-123. doi:10.1377/hlthaff.2019.01088 31821045

[27] Sugar S, Peters C, De Leew N, Sommers BD. Medicaid churning and continuity of care: evidence and policy considerations before and after the COVID-19 pandemic. Issue Brief No. HP-2021-10. Office of the Assistant Secretary for Planning and Evaluation, U.S. Department of Health and Human Services. April 12, 2021. Accessed November 1, 2024. https://aspe.hhs.gov/sites/default/files/private/pdf/265366/medicaid-churning-ib.pdf

[28] Conderino S, Bendik S, Richards TB, . The use of electronic health records to inform cancer surveillance efforts: a scoping review and test of indicators for public health surveillance of cancer prevention and control. BMC Med Inform Decis Mak. 2022;22(1):91. doi:10.1186/s12911-022-01831-8 35387655 PMC8985310

[29] Kruse CS, Stein A, Thomas H, Kaur H. The use of electronic health records to support population health: a systematic review of the literature. J Med Syst. 2018;42(11):214. doi:10.1007/s10916-018-1075-6 30269237 PMC6182727

[30] Ross MK, Sanz J, Tep B, Follett R, Soohoo SL, Bell DS. Accuracy of an electronic health record patient linkage module evaluated between neighboring academic health care centers. Appl Clin Inform. 2020;11(5):725-732. doi:10.1055/s-0040-1718374 33147645 PMC7641664

[31] Devine EB, Totten AM, Gorman P, . Health information exchange use (1990-2015): a systematic review. EGEMS (Wash DC). 2017;5(1):27. doi:10.5334/egems.249 29881743 PMC5983051

[32] Payne TH, Lovis C, Gutteridge C, . Status of health information exchange: a comparison of six countries. J Glob Health. 2019;9(2):0204279. doi:10.7189/jogh.09.020427 31673351 PMC6815656

[33] Bhattacharyya O, Rawl SM, Dickinson SL, Haggstrom DA. Comparison of health information exchange data with self-report in measuring cancer screening. BMC Med Res Methodol. 2023;23(1):172. doi:10.1186/s12874-023-01907-7 37491208 PMC10367403

