# Supplementary material for: Delivering Guideline-Concordant Care for Patients With High-Risk HPV and Normal Cytologic Findings
Source: JAMA Netw Open. 2025 Jan 17;8(1):e2454969. doi: 10.1001/jamanetworkopen.2024.54969 (PMC11742536; doi:10.1001/jamanetworkopen.2024.54969)
Supplement: Supplement. — Data Sharing Statement [file jamanetwopen-e2454969-s001.pdf]

Data Sharing Statement

Tiro. Delivering Cervical Cancer Management Guidelines for Patients With High-Risk Non–16/18 Genotype Human Papillomavirus and Normal Cytologic Finding. *JAMA Netw Open.* Published January 17, 2025. doi:10.1001/jamanetworkopen.2024.54969

Data
Data available: No

Additional Information
Explanation for why data not available: As part of the NCI-funded PROSPR METRICS Consortium, we have a mechanism to receive, review, and approve data sharing requests. Data will be made available to researchers with adequate resources to cover the regulatory and data sharing costs. Data will be made available after approval of a concept proposal aligned with current data approvals, and with a signed data access agreement. Please email the corresponding author to initiate a request.
